# Pilot implementation of allied health assistant roles within publicly funded health services in Queensland, Australia: results of a workplace audit

**DOI:** 10.1186/1472-6963-14-258

**Published:** 2014-06-16

**Authors:** Michelle Stute, Andrea Hurwood, Julie Hulcombe, Pim Kuipers

**Affiliations:** 1 Metro North Hospital and Health Services, Herston, Queensland, Australia; 2 Allied Health Professions Office, Herston, Queensland, Australia; 3 Metro South Hospital and Health Services & CONROD, Griffith Health Institute, Griffith University, Meadowbrook, Queensland, Australia

## Abstract

**Background:**

Allied health assistants provide delegated support for physical therapists, occupational therapists and other allied health professionals. Unfortunately the role statements, scope of practice and career pathways of these assistant positions are often unclear. To inform the future development of the allied health assistant workforce, a state-wide pilot project was implemented and audited.

**Methods:**

New allied health assistant positions were implemented in numerous settings at three levels (trainee level, full (standard) scope and advanced scope level). Six months after implementation, 41 positions were audited, using a detailed on-site audit process, conducted by multiple audit teams.

**Results:**

Thematically analysed audit findings indicated that both the full (standard) scope and the advanced scope positions were warranted, however the skills of the allied health assistants were not optimally utilised. Contributing factors to this underutilization included the reluctance of professionals to delegate clinical tasks, inconsistencies in role descriptions, limitations in training, and the time frame taken to reach an effective skill level.

**Conclusions:**

Optimal utilisation of assistants is unlikely to occur while professionals withhold delegation of tasks related to direct patient care. Formal clinical supervision arrangements and training plans should be established in order to address the concerns of professionals and accelerate full utilisation of assistants. Further work is necessary to identify the key components and distinguish key features of an advanced allied health assistant role.

## Background

In Queensland, Australia, some allied health professional (AHP) positions (including physical therapists, occupational therapists, speech pathologists, dietitians, medical radiation professionals, social workers, pharmacists and podiatrists) are supported by allied health assistants (AHAs). These AHAs work under the delegation and supervision of AHPs to assist in the provision of allied health services. While some AHAs work within one discipline area (e.g. physical therapy assistant), many work across a few disciplines, providing assistance to multiple AHPs. Since they work under supervision and delegation, they are an unregulated workforce, usually trained ‘on the job’, or through completion of a vocational certificate [1].

Allied health assistants are potentially key members of health care teams who can contribute to better patient outcomes and help manage demand for allied health services [2-5]. The support of AHAs may release AHPs to undertake more high level tasks, allow more AHP treatment time and enhance access to services for clients. Increasing skill shortages across the health workforce necessitate that the capabilities of all team members should be used optimally [6]. For AHPs, this may mean that some of their tasks should be delegated to an AHA who has relevant training and adequate supervision.

Altering the skills mix of healthcare teams through increasing the role of assistants is reported to have benefits for patients, AHPs and the healthcare system. For example, a study on the appointment of trained diatry assistants noted shorter waiting times and greater availability of podiatrists for patient care [4]. Likewise, a study on the use of dietetic assistants noted better clinical outcomes for patients [5], and a recent report has linked the use of assistants with the provision of more community-based care [7]. For AHPs it has been noted that the appointment of AHAs allows them to focus on patients with more complex needs, commensurate with their level of training [8]. Further, health services are reported to benefit through being able to provide a more cost effective and productive service [9].

The extent of AHA involvement in direct patient care typically depends on a number of variables including the nature of the relationship between the assistant and the delegating professional(s), the AHA’s skills and attributes, and the work context [10]. For AHAs, clarity of role and scope of practice is vital, particularly within large teams and across diverse settings [11-13]. Vague role delineation lowers morale and erodes respect for AHAs [14]. Lack of clear delineation also hinders role development and results in ineffective utilisation [15], and inappropriate tasks [16,17].

To date, in Queensland public sector health services, all AHAs have been salaried at the same level, despite variations in duties and skills. There have been no formal entry level positions, opportunities for career advancement or articulation with professional roles. As noted elsewhere [18], such flat career structures can be problematic, promoting boredom or job dissatisfaction and giving assistants cause to move to other positions offering greater opportunities.

In 2007, a non-degree vocational training “Certificate IV in Allied Health Assistance” was introduced in Australia [19]. Currently many AHAs are trained through both formal (for example, the Certificate IV), and informal means (for example, in-service training or ‘shadowing’).

Internationally there has been interest in extending standard AHA functions towards advanced scope of practice positions. In such positions AHAs may have the ability to act autonomously, have primary contact status, plan care programs, and discharge clients [20-23]. In Australia these advanced level positions have not been prominent, despite calls for general expansion of AHA roles [8].

Across allied health literature there are diverse perspectives regarding the burgeoning role of AHAs. Whilst some authors have highlighted the benefits of using assistants to undertake routine tasks and less complex assessments [6,24,25], others have argued that the use of assistants can invalidate and blur the role of professionals [7,26,27]. Clearly this is an important and potentially contentious area which requires careful implementation and evaluation. In response, this pilot study was undertaken on a state-wide basis, across multiple sites, to explore utilisation of the skills of AHAs within publically funded Queensland health services. The current paper describes the process of testing and evaluating new AHA positions at trainee level, full scope of practice (standard AHA level), and advanced scope of practice level. Key differences between these different levels of position are noted in Table 1. The pilot study also sought to explore whether greater role clarity would improve utilisation of AHAs at the different levels, highlight potential aspects of AHA career pathways, and inform their training and supervision arrangements.

**Table 1:** Key differences between three AHA levels

	**Clinical decision making**	**Supervision and delegation**	**Typical qualifications**
**Trainee AHA**	Not part of role	Delegated role. Works under direct supervision from AHP	‘On the job’ training
**Full (standard) scope AHA**	Minimal (Frequently seen patient groups/conditions with noncomplex presentations, according to protocol)	Delegated role. Works under direct or indirect supervision from AHP	Certificate III or Certificate IV in Allied Health Assistance
**Advanced scope AHA**	Some (Frequently seen patient groups/conditions, with more complex presentations, according to protocol).	Delegated role. Works mostly under indirect supervision from AHP	Certificate III or preferably Certificate IV Allied Health Assistance

## Methods

As described elsewhere [28], generic role descriptions for AHAs were developed across 13 allied health professions, through a combination of focus groups, a Delphi survey, and the analysis of existing role descriptions. The majority of these role descriptions were generic in nature (in order to promote role consistency), however in the case of medical imaging, pharmacy and social work, they were contextualised to better fit the particular duties required of assistants in those settings. The current analysis aimed to evaluate the scope of practice of AHAs as documented in the generic and contextualised role descriptions across the three levels to which AHAs were appointed in Queensland, namely, trainee AHA, full (standard) scope AHA, and advanced level AHA.

### AHA pilot study positions

Within this service model development initiative, 51 AHA trial positions were established at the three levels (Table 2). Criteria for selection of demonstration pilot project sites included, health services in regions of high population growth, in which there was documented increasing demand for services, and in which it was recognised that the current model of care was unsustainable. Demonstration projects commenced in August 2009, and were a mixture of rural, regional and large metropolitan hospitals, rehabilitation facilities and home based care, across nine sites. This diversity of sites and settings was deliberate in order to explore the viability and outcomes of employing AHAs at a state-wide level.

**Table 2:** Setting, location and discipline of 51 (audited and non-audited) AHA roles

**Audited roles (n = 41)**															
**Level**		**Setting**			**Location**			**Discipline**							
	**Mean duration - months**	**Acute**	**Sub-acute**	**Community**	**Metropolitan**	**Regional**	**Rural**	**Multidisciplinary**	**Occupational therapy**	**Physiotherapy**	**Speech pathology**	**Pharmacy**	**Social work**	**Dietetics & Nutrition**	**Medical imaging**
**Trainee**	7.0	1	1		2			1	1						
**Full (standard) scope**	7.2	16	1	7	20	1	3	10	3	6	2	1	1		1
**Advanced scope**	6.0	9		6	8	5	2	8	2	3		1		1	
**Totals (Mean)**	**(6.7)**	**26**	**2**	**13**	**30**	**6**	**5**	**19**	**6**	**9**	**2**	**2**	**1**	**1**	**1**
**Non-audited roles (n = 10)**															
**Level**		**Setting**			**Location**			**Discipline**							
	**Mean duration - months**	**Acute**	**Sub-acute**	**Community**	**Metropolitan**	**Regional**	**Rural**	**Multidisciplinary**	**Occupational therapy**	**Physiotherapy**	**Speech pathology**	**Pharmacy**	**Social Work**	**Dietetics & nutrition**	**Medical imaging**
**Trainee**	6.0	1			1					1					
**Full (standard) scope**	5.4	5			2	2	1			1	1	1			2
**Advanced scope**	1.7	2		2	2	2		2		1		1			
**Totals (Mean)**	**(4.4)**	**8**		**2**	**5**	**4**	**1**	**2**		**3**	**1**	**2**			**2**

Allied health professionals at each of the selected sites analysed their respective activities and roles to identify tasks that could be safely delegated to an assistant. Through these processes, task lists were developed for each of the 51 trial positions.

AHAs were recruited to the positions using the role descriptions and task lists. In cases where candidates did not already possess a Certificate IV in Allied Health Assistance, they were financially supported and encouraged to acquire this qualification before and during the project. Additional ‘on the job’ training was provided locally.

Pilot study sites were provided with general guidelines on supervision, management and delegation. Tools available to all sites included: templates for documenting supervision, a presentation on supervision and management, two self directed learning modules on supervision of assistants, a detailed list of standard tasks appropriate for each level, an orientation package and clinical documentation guidelines for assistants. Pilot study sites were encouraged to prepare localised induction and training plans for the positions.

### Role audits

At the end of the pilot trials, 41 positions were audited over a two-month period. As described below, 10 positions were not included in the audit due to staff turnover. The audit process specifically devised for this pilot was detailed in nature, inclusive of multiple perspectives, and deliberately focused on each local worksite.

The audit team consisted of 16 Queensland Health allied health professionals with relevant clinical experience, recent practice supervising AHAs, and an understanding of issues surrounding delegation and auditing processes. Auditors received training in the use of specifically developed audit tools to ensure consistency across the audits. Audits were conducted by pairs of auditors who were external to the pilot trial sites, and who visited each site for two consecutive days. While this audit method was relatively time- and cost-intensive, it was seen as a rigorous way of obtaining detailed data on the pilot trial of these AHA positions.

At least one of the auditors in each pair had a qualification and/or experience directly relevant to each position they audited. Auditors used a variety of data collection methods including document review (for role descriptions, task lists, induction and training plans, competency assessment, supervision agreements and clinical documentation), as well as task observation. They also conducted interviews with all of the 41 AHAs, with each of their respective supervisors, and in each case, with members of their clinical team (Additional file 1).

To enhance objectivity and rigour, the audit tools consisting of a data collection workbook and summary document were developed by an external agency. The tools, which provided the structure for the audit, focussed on the subjects of: role descriptions, the task lists, induction and training, as well as supervision and delegation. The 13 questions addressing these subject areas are noted in Table 3, and the process is documented in Additional file 1.

**Table 3:** Focus questions for audit data collection and analysis

**Subject**	**Focus questions**
Role descriptions	Do the generic and contextualised role descriptions have transferability to a variety of worksites, disciplines, clinical areas and locations?
	Do they promote consistency in the role and scope of practice through supporting development of task lists?
	Are the key accountabilities clear, appropriate and do they differentiate roles at different levels?
Task lists	Do the tasks align with the key accountabilities at each level?
	Should additional tasks be added to the list?
	Were all the tasks on the list being delegated to the assistant? If not, why?
	Were there any tasks being delegated to the assistant that were not on the task list? What were they?
	Did the task list describe the required level of supervision for each role?
Induction and training	Was there a process in place to ensure that each allied health assistant was competent to perform their role?
Supervision and delegation	Were the allied health assistant and the delegating allied health professionals aware of the assistant’s scope of practice?
	Were formal supervision arrangements in place?
	Were all tasks that should have been delegated to the assistant being delegated?
	Was the assistant working without appropriate supervision or performing tasks that should not have been delegated to them (due to skill deficiencies or client complexity for example)?

### Analysis

The foundation for analysis of audit data comprised standardised and consistent data collection facilitated by a workbook. The use of two auditors for each position provided a degree of objectivity to the data collection and a means of corroborating the data and scoring system. Further, as noted in Additional file 1, the use of two project officers to independently collate and thematically analyse the data, added rigour and reliability to the analysis. In instances of discrepancy in analysis were recorded and discussed by the project officers with each noting their reasons and arriving at an agreed consensus. Themes were prioritised according to how frequently they were identified, but also with reference to the importance assigned by the auditors. Consistent themes were collated and linked with the respective evaluation questions by the project officers. Representative examples of audit summary data pertaining to each theme are noted in Table 4. The linked questions and themed data comprised outcomes of the pilot trial. Research team members and one of the project officers then met to establish consensus on editing and finalising the role descriptions based on these outcomes, and to make recommendations for future service models (Additional file 1). As an investigation of an evaluation and audit for which reports and results are in the public domain, ethics committee approval was not required by management.

**Table 4:** Themes and representative quotes (with type of AHA position)

**Theme**	**Representative quote – Recorded observation by Audit Team**
Underutilisation of AHAs due to:	
• Limited understanding of the scope of the AHA role or knowledge of AHA tasks.	“It appears that there is clarity required (consistency) around what is ‘in scope’ for a Social Work Assistant (SWA) role - this requires significant further discussion and input from all team members” *(Full (standard) Scope: Metropolitan, Discipline-specific, Hospital)*
• Limited time for AHA training and skill development	*“*Some of the duties require more training - so are not being performed yet, but may be in future” *(Full (standard) Scope: Regional, Multidiscplnary, Hospital)*
• Unwillingness of AHP to delegate to AHA	“AHP withheld some tasks perceived to be inappropriate for AHA. Training in supervision and delegation to assistants would be helpful”. *(Advanced scope: Rural, Multi-disciplinary, Community)*
• Insufficient analysis of AHP role to determine tasks that could be safely delegated.	“Needs further definition of the task requirement and some structure around delegation of ‘when’ [it is] appropriate for the OTA to be delegated this task “. *(Advanced Scope: Metropolitan, Discipline-specific, Hospital)*
• Insufficient confidence/or skills on part of the AHP to delegate effectively.	“AHP are not satisfied that the AHA has had enough exposure/experience to complete this task yet without supervision” *(Full (standard) scope: Remote, Multi-disciplinary, Hospital).*
• Lack of an established relationship or confidence in the AHA	“Some duties have been performed … but since the current OT has begun [these tasks] have been ceased either due to AHA feeling they didn’t have sufficient competency or OT feeling it wasn’t in the AHA’s scope*”. (Full (standard) scope: Regional, Multi-disciplinary, Hospital)*
Advanced level exists in practice – some AHAs are working independently with relatively complex patients.	“[The advanced AHA ] Identifies and conducts quality improvement activities…with guidance and prompting from supervising AHP, simple ideas can be initiated into improvements in processes ” *(Advanced scope: Metropolitan, Discipline-specific, Hospital)*
Contextualised role descriptions more accurately reflect duties than generic role descriptions	“Physio is OK, OT and SP not to full scope. Duties statement needs revising and rewording. Difficulties with having a multi-disciplinary role. Maybe discipline-specific would work better”. *(Full (standard) scope: Metropolitan, Multi-disciplinary, Hospital)*
‘On the job’ training, as part of a formal qualification or not, is the most appropriate and accessible form of training	“[The] AHP reported a high level of training and supervision was required and provided to support skill/task development [in the AHA]*” (Advanced scope: Regional, Discipline-specific, Hospital)*
Relatively few of the evaluated AHA roles had a formal training plan in place	“[There was] limited training provision for AHA due to the isolated location and no structured training plan”. *(Full (standard) scope: Remote, Multi-disciplinary, Hospital)*
Certificate IV was insufficient training for advanced scope roles	“Cert IV not enough for advanced role - needs higher level training”. *(Advanced scope: Regional, Multi-disciplinary, Community)*
AHAs reported the amount of formal supervision from AHPs was inadequate	“[AHA] reported … formal supervision has predominately centred around the Cert IV training and achievement of competencies which [she] felt was not adequate to continue her professional growth” *(Full (standard) scope: Discipline-specific, Metropolitan, Hospital)*
It takes 6 months for AHAs to reach effective skill level. Longer for trainees and advanced scope roles.	“[The AHA took] a long time to train (more than 6 months). Informal training process was ad-hoc. More formal supervision would be of benefit. AHP’s confidence in AHA [is] low” *(Full (standard) scope: Metropolitan, Discipline-Specific, Hospital)*

## Results and discussion

Quantitative and qualitative findings drawn from the audit conducted by 16 allied health professionals are provided below. As presented in Table 2, 51 positions were trialled across a range of geographic regions, clinical areas and levels. Twenty seven of these positions were newly recruited for the pilot project, and 24 were redesigned or upgraded from existing positions to align with the pilot trial. The duration of the positions varied, with 22 trialled for less than 6 months, and 29 positions being trialled for between 7 and 9 months.

As reflected in Table 2, 41 of these positions, including two trainee positions, 25 full (standard) scope of practice positions and 14 advanced scope positions were audited by the teams. Ten positions were not audited because they were vacant at the time of the audit, or had recently been filled and the incumbents were still becoming established in the position. The mean duration of these 41 positions at time of audit was over six months, (range 2–9 months) and well over half were in acute and metropolitan settings.

### Tasks undertaken

The audit documented that only 56% of AHA roles (23 positions) had clear and comprehensive task lists that specified the level of complexity and autonomy required for the positions. As a consequence, half of the incumbents (21 positions) were found to be performing duties that were not recorded on the task list. This issue has been previously noted [16] as problematic in implementation of assistant positions, and was seen as a key issue to be addressed.

Some of the additional duties identified in the audit included case conferences, staff meetings, equipment maintenance, administration backfill and patient transport. While performing a number of support functions is fundamental to AHA positions [29], auditors found that AHAs performed a greater portion of non-clinical duties (particularly administration) than would be expected for a role intended to primarily focus on direct patient care. Auditors found that 22% of assistants (9 positions) in trial roles were performing tasks for which they had not been adequately trained. Review of the audit reports indicated that this was often due to insufficient time for training within the brief time frame of the project. However in some cases AHAs performed such tasks as a result of inappropriate delegation of duties, being assigned tasks which were too complex for the level of position.

### Scope of role and delegation

Auditors noted that almost half of the incumbents (46%, 19 positions) were not working to the full scope of their position, despite having been in the trial positions for an average of over 6 months. Qualitative findings indicated that in some teams there was limited understanding of the scope of the trial positions and insufficient time for training, which resulted in limited opportunities for assistants to gain experience in all aspects of their role. The audit revealed that while assistants in advanced level positions mostly performed tasks equivalent to full (standard) scope positions, only a third of their time was spent performing more complex advanced level tasks. Based on audit interviews, this relative underutilisation of advanced level assistants may be attributed to a number of factors including ambiguity in duty statements or the unwillingness of professionals to delegate more complex tasks (despite those tasks being recorded on the task list). Interestingly while recent studies have noted numerous concerns with greater use of assistants [30,31], they do not appear to have recognised underutilisation and the practical implications of underutilisation in these positions.

In some cases AHPs did not sufficiently analyse their activities or the respective patient clinical pathways to identify tasks that could be permanently delegated to an advanced assistant. In a few cases, because these tasks were not frequently performed by the professional, they did not bother to delegate them. Even though AHAs were generally underutilised, audit findings indicated that in a number of settings, advanced level AHAs were successfully working at a considerable level of independence, and with relatively complex patients. Further, some were assisting with conducting education sessions, and making decisions on patient care plans according to the protocol and within their delegated role. Based on these observations and findings, and the opinions of workplace supervisors, it was concluded that there was sufficient differentiation between the full (standard) and advanced scope of practice positions to warrant two separate roles in practice.

Based on data obtained by the auditors, it would appear that in more than half of the trial roles (61%, 25 positions), allied health professionals’ withheld delegation of clinical tasks to assistants. While this issue has been generally noted in the broader assistant literature [31,32], the current practice-based audit in an allied health setting documented a number of dimensions. Audit summaries indicated that the professional’s readiness to delegate was related to: (a) their familiarity with the task list, (b) the quality of their relationship with the assistant, (c) their confidence in the assistant and, (d) their belief about whether the task was appropriate to be delegated to an assistant.

Further, some professionals expressed concern about expansion of the assistant’s scope of practice, when as a delegated function, the professional remains accountable. In some instances, professionals believed that such tasks should be retained within their scope of practice; and some had insufficient experience, skills, and knowledge to confidently delegate responsibilities. From the perspective of assistants, some were not adequately trained to perform more complex tasks due to insufficient time, competing demands, and because there was no formal training for the advanced level role. Audit interviews confirmed previous research noting reluctance to delegate [22,29] and found that in this sample, professionals delegated less when they didn’t know the assistant or lacked confidence in their ability. Likewise, in cases of staff turnover or when professionals were unfamiliar with the task list, delegation was limited.

These findings go beyond current literature by highlighting potential areas to direct training for AHAs and professionals. Clearly it is important for all assistants to achieve appropriate levels of competency. It is also important that all professionals have adequate understanding of the importance of delegating, skills in delegation, and confidence in the structures of delegation. Greater optimisation of assistants may be achieved if the process is based on skills and confidence rather than relying on established relationships [31].

### Role descriptions

Based on audit findings, 42 of 49 (85%) of the key accountabilities in the generic role descriptions required changes, compared with 11 of 53 (20.7%) of the key accountabilities in the contextualised role descriptions (used for pharmacy, medical imaging and social work). These findings provide an interesting practice-based confirmation of studies which have identified unclear role descriptions and blurred role boundaries as problematic [29]. Feedback obtained in the current audit indicated these changes to the generic role descriptions were necessary because the generic language used was not always relevant to the discipline, clinical area, or geographical location. An example of how key accountabilities were contextualised is provided in Table 5. Further, respondents indicated that the level and nature of professional supervision should be more clearly described for each role.

**Table 5:** Example of generic and contextualised role descriptions (communication and referral)

**Generic AHA role description**	**Pharmacy assistant role description**
Refer to and liaise with health care providers within the immediate team as well as community services using decision support tools, clinical pathways and patient specific guidelines.	Refer to and liaise with health care providers within the immediate team as well as community health providers such as community pharmacists and general practitioners, under the delegation of a pharmacist.

Audit data indicated that the contextualised role descriptions more accurately reflected the duties than did the generic ones. While generic role descriptions promoted some consistency in the role and scope of practice across disciplines, clinical areas and geographic locations, feedback from staff audited suggested that their value was limited when they were too generic. A number of allied health professionals reported difficulties with interpretation of role terminology across disciplines. Tailored role descriptions such as those used in this study for medical imaging, social work and pharmacy were found to promote role standardisation with greater clarity while still enabling transferability to various clinical areas and locations within the discipline. This is consistent with recent studies which have recommended more structure and greater clarity in AHA role descriptions [33].

Despite the positive judgements of specialist assistant positions reported in interviews, the audit data did not demonstrate more appropriate use of the skills of these AHAs compared with those under generic role descriptions. This was a surprising finding. It would appear that while tailored role descriptions are important, optimal utilisation of these positions is a function of factors beyond the written description [31].

### Induction and training

As noted in a recent systematic review [30], the training and induction of assistants is a highly important but contentious area. In the current audit, on the job training, whether delivered as part of a formal qualification or not, was identified as the primary source of training for the trial positions. Seventy six percent of assistants (31 positions) in trial positions had either completed or were enrolled in a Certificate IV Allied Health Assistance. Additionally, 85% (35 assistants) reported that in-service training and activities had an important function in training them for their role, and 27 (66%) said the same of other formal training courses. Auditors found that the time required to train the AHAs to be effective in their positions varied from 1–3 months for those in full (standard) scope roles to 3–6 months for assistants in trainee roles and those in advanced roles.

Recognising that all of the pilot trial positions were new or redesigned, relatively few (41%, 17 positions) had a formal training plan in place. This was identified as another issue of concern. Auditors noted that this was attributed to the short time frame of the trial, that training requirements for the new roles had not been determined, and a preference for informal training on an ad-hoc basis. However, if such plans had been established at commencement and reviewed regularly they may have substantially reduced the amount of time that the incumbents took to perform effectively in their positions. Audit findings suggested that “on the job” training, whether part of a formal qualification or not, remains the most appropriate, accessible and relevant form of training for AHAs within publically funded health services in Queensland.

While the Certificate IV Allied Health Assistance was seen as the most relevant qualification for assistants within Queensland publically funded health services, audit results suggested it was insufficient for assistants working at an advanced scope of practice level. This may also have impacted effectiveness and delegation, and is an issue for future consideration of advanced level roles. From the present audit, and in light of issues identified in the literature [30], it is clear that there were a number of inconsistencies in the training of AHAs.

### Supervision

Audit data also revealed that 28 assistants (68%) in trial positions received formal one-on-one supervision. Despite this, almost half of audited positions (46%, 19 positions) reported that this was inadequate, due to the infrequency of the sessions or the limited experience of the assistant. Findings suggest that supervising professionals underestimated the level of formal supervision required by incumbents in trial positions. While most had formal supervision arrangements, nearly half of the assistants described them as inadequate for their needs. Recognising that many AHAs feel unprepared for their roles [30], more comprehensive supervision arrangements would have been preferable. This was also identified as an issue for future consideration in the implementation of AHA positions.

### Time frame

The audit noted that nearly half of the assistants were not working to the full scope of their role at the time of audit. It was noted that in the case of a redesigned role, it takes at least three months, and in the case of a new role it takes approximately six months for an AHA to reach an effective skill level. Further, it appeared that trainee positions (which recruited inexperienced staff) and advanced level positions (for which there was no formal training available) were comparatively slow in demonstrating efficacy. Some full (standard) scope roles (which closely resembled existing roles with which professionals were familiar) reached effective skill levels comparatively quickly. This may have impacted on findings in the current short duration trial, and has implication for planning the time frame of future trials.

### Limitations of this study

Based on data analysis and debriefing with auditors on completion of the audit, it appeared that the audit tools while generally useful, lacked sensitivity. This may have limited the potential interpretation of the data, particularly in terms of differentiating between full (standard) and advanced scope positions. For example, the tools adequately captured how frequently a task was performed, but not how much time was spent on each task. Similarly, the tools did not result in sufficient detail about task complexity and level of supervision for the audit team to reliably assess the appropriate level of the position.

While the diversity of sites, locations, service settings and professional contexts was part of the intended rationale of this pilot project on a state-wide level, it may have also clouded results that may have relevance to one context or setting (positively or negatively). Likewise the focus in this study on general themes, may have obscured variation in the data across contexts or settings. As such, these findings should be taken as a general indication, and should form the basis for more targeted future research.

## Conclusions

This study tested three generic role descriptions for AHAs and concluded that generic role descriptions can be problematic if issues such as ambiguous wording and particular uses of terminology cannot be overcome. Role descriptions tailored to individual disciplines would appear to promote role consistency while also being transferable across clinical areas and geographical locations. To assist AHAs to be effective in their positions, trials of new or redesigned roles should be of at least 6 months duration, with formal supervision arrangements and a training plan established from the outset.

It was concluded that while there was sufficient differentiation between full (standard) and advanced roles for an advanced scope position to exist within publically funded health services in Queensland, further work needs to be done to ensure that advanced scope AHAs are utilised to their full extent. For this to operate effectively in the future, the concerns and attitudes of professionals should be addressed and suitable training plans should be put in place. Likewise, professional and registration bodies require clarity, security and confidence in the scope of their own positions to proactively deal with such workforce reform.

## Competing interests

The authors declare that they have no competing interests.

## Authors’ contributions

MS was the main project officer and co-authored the initial manuscript. JH initiated the project, analysed data and co-authored the initial manuscript. AH managed the project, analysed data and co-authored the initial manuscript. PK assisted with final data analysis, interpretation and drafted the final manuscript. All authors contributed to, read and approved the final manuscript.

## Pre-publication history

The pre-publication history for this paper can be accessed here:

http://www.biomedcentral.com/1472-6963/14/258/prepub

## Supplementary Material

Additional file 1 Summary of the audit process, methods, analysis and tools. Click here for file
