# Supplementary material for: Pilot implementation of allied health assistant roles within publicly funded health services in Queensland, Australia: results of a workplace audit
Source: BMC Health Serv Res. 2014 Jun 16;14:258. doi: 10.1186/1472-6963-14-258 (PMC4074147; doi:10.1186/1472-6963-14-258)
Supplement: Additional file 1 — Summary of the audit process, methods, analysis and tools. [file 1472-6963-14-258-S1.docx]

Additional File 1. Summary of the audit process, methods, analysis and tools

| **Process** |  | **Tools (which comprised the audit workbook) and methods** |  |
| --- | --- | --- | --- |
| Identify the position to be audited |  | Documentation of title of position, level of position, new or redesigned role, qualifications held by AHA and delegating AHP, number of AHAs in similar position, number of AHAs who were audited, names of auditors and audit dates. |  |
|  |  |  |  |
| Review of duties statement for the role |  | Through interviews, document review and/or observation, each task on the duties statement is analysed in a table to determine:   - If the task is indeed being performed, - How frequently it is being performed, - Whether the task is clinical or non-clinical in nature - If it is not being performed, why? |  |
|  |  |  |  |
| Document review |  | All available documentation on the role is reviewed and key observations documented in a table, in the following areas pertaining to the role:   - Orientation and induction - Training and competency - Delegation and supervision arrangements - Clinical documentation in patient files |  |
|  |  |  |  |
| Semi-structured stakeholder interviews |  | Prompt questions are used to guide: interviews with AHAs, interviews with AHPs, and interviews with the multidisciplinary teams in which the AHA trial position is located. These prompt questions focus on:   - duties, - induction, - training, - management of delegation and supervision and - general impressions.   Notes from interviews are transcribed into a table against each of the prompt questions. |  |
|  |  |  |  |
| Review of role descriptions |  | Through interviews, observation and document review, auditors review key accountability statements in each role description, documenting in a table:   - Does the key accountability statement clearly describe what is expected of a person performing this role? - If it does not, what are the reasons for this? - What is the evidence, or tasks inconsistent with the key accountability statement? - How might the key accountability statement be changed to better describe the actual role? |  |
|  |  |  |  |
| Analysis |  | Analysis comprising: (a) thematic categorisation of data,  (b) prioritisation of findings (using frequency of reporting and qualitative strength of evidence),  (c) collation of consistent themes, and  (d) linking of themes with original evaluation questions to inform conclusions. | |
|  |  |  | |
| Conclusions & Recommendations |  | Key conclusions and recommendations drafted. | |
